# Gene and protein patterns of potential prion-related markers in the central nervous system of clinical and preclinical infected sheep

**DOI:** 10.1186/1297-9716-44-14

**Published:** 2013-03-11

**Authors:** Hicham Filali, Enric Vidal, Rosa Bolea, Mercedes Márquez, Paola Marco, Antonia Vargas, Martí Pumarola, Inmaculada Martin-Burriel, Juan J Badiola

**Affiliations:** 1 Centro de Investigación en Encefalopatías y Enfermedades Transmisibles Emergentes. Facultad de Veterinaria, Universidad de Zaragoza, Zaragoza, Spain; 2 PRIOCAT Laboratory, Centre de Recerca en Sanitat Animal (CReSA), UAB-IRTA, Campus de la Universitat Autònoma de Barcelona, Bellaterra, Barcelona, Spain; 3 Laboratorio de Genética Bioquímica (LAGENBIO), Facultad de Veterinaria, Universidad de Zaragoza, Zaragoza, Spain

## Abstract

The molecular pathogenic mechanisms of prion diseases are far from clear. Genomic analyses have revealed genetic biomarkers potentially involved in prion neuropathology in naturally scrapie-infected sheep, a good animal model of infectious prionopathies. However, these biomarkers must be validated in independent studies at different stages of the disease. The gene and protein expression profiles and protein distribution of six potential genetic biomarkers (i.e., *CAPN6*, *COL1A2*, *COL3A1, GALA1, MT2A* and *MTNR1B*) are presented here for both the early and terminal stages of scrapie in five different brain regions. Gene transcription changes were confirmed in the medulla oblongata, and the expression profiles were generally similar in other central nervous system regions. The changes were more substantial in clinical animals compared to preclinical animals. The expression of the CAPN6 protein increased in the spinal cord and cerebellum of the clinical and preclinical brains. The distribution of the GALA1 was identified in glial cells from the cerebellum of scrapie-infected animals, GALA1 protein expression was increased in clinical animals in the majority of regions, and the increase of MT2A was in agreement with previous reports. The downregulation of MTNR1B was especially marked in the Purkinje cells. Finally, although collagen genes were downregulated the protein immunostaining did not reveal significant changes between the scrapie-infected and control animals. In conclusion, this study of gene transcription and protein expression and distribution confirm CAPN6, GALA1, MTNR1B and MT2A as potential targets for further prion disease research.

## Introduction

Transmissible spongiform encephalopathies (TSE), or prion diseases, are a group of fatal neurodegenerative disorders of sporadic, genetic or infectious origin [1]. This group of disorders includes Creutzfeldt–Jakob disease (CJD), Gerstmann-Sträussler-Scheinker disease, kuru and fatal familial insomnia in men [2-4], scrapie in sheep and goats [5] and bovine spongiform encephalopathy (BSE) in cattle [6]. There is compelling evidence [7] that, according to the “protein-only” hypothesis [8], TSE are caused by the conversion of normal mammalian prion protein (PrP^c^) into its pathological conformation, or scrapie-associated prion protein (PrP^Sc^), which is abnormally folded, β-sheet enriched and protease resistant [9]. The accumulation of PrP^Sc^ in the central nervous system (CNS) induces neurodegeneration, spongiosis and glial cell activation and death [1,8,10,11]. Prions are naturally infectious by the oral route and can be transmitted experimentally by inoculation. However, the long incubation period of these diseases has slowed the progress of research investigation.

Classical scrapie in sheep is the earliest known prionopathy. The first descriptions date back to 1732 [12], and infected animals are considered to be a good animal model of the infectious prion diseases. To understand the pathogenesis of scrapie and other prion diseases, a large number of studies have been performed, including the analysis of gene and protein expression in both the early and terminal stages of the disease [13-19]. This research is of critical importance to identify not only diagnostic markers but also possible therapeutic targets of this incurable group of diseases.

We recently published [20] the gene transcription profiles from the medulla oblongata of sheep, which were naturally infected with clinical scrapie, using a microarray platform. In this study, criteria based on fold-change values, gene ontology and association analyses with PrP^Sc^ deposition, astrocytosis and spongiosis were used to select a set of neuropathology-related genes that had previously been reported to be associated with prion and other neurodegenerative diseases in experimental models [21-26]. The gene transcription results were confirmed by quantitative Real Time PCR (qRT-PCR) using the same animals that were included in the microarray study. The gene transcription data can yield relevant information regarding ongoing processes in the diseased brain, but the role of each individual gene requires further investigation. An evaluation of whether the gene transcription changes reflect the levels of the end-product of each gene (i.e., the protein itself), and the determination of the cellular locations of these proteins may help to elucidate the biological relevance of the differential gene transcription observed between healthy and scrapie-infected sheep.

In the present study, in the medulla oblongata of a larger set of clinical naturally infected sheep, we confirm the differential expression of the six genes that displayed the largest up- or down-regulation in our previous work [20]: three upregulated (i.e., calpain 6 [*CAPN6*], galanin 1 [*GALA1*] and methallothionein 2A [*MT2A*]) and three downregulated genes (i.e., collagen 1 alpha 2 [*COL1A2*], collagen 3 alpha 1 [*COL3A1*] and melatonin receptor 1b [*MTNR1B*]). We also extended the analysis to include gene transcription data from three additional CNS regions. In addition, we evaluated whether these changes are present in the preclinical stages of the disease. Finally, for the first time, we report the distribution of the encoded proteins in different regions of the CNS of both clinical and preclinical field cases of ovine scrapie.

## Materials and methods

### Ethics statement

This study was approved by the Ethics Committee for Animal Experiments of the University of Zaragoza (Permit Number: PI02/08) and was carried out in strict accordance with the recommendations for the care and use of experimental animals and in agreement with the national law (R.D. 1201/2005).

### Animals

Scrapie animals obtained from scrapie-infected flocks from several geographical regions were maintained for research purposes at our research center. The animals were genotyped for *PRNP* polymorphisms. The complete PRNP gene open reading frame was sequenced as previously described [27], and the sheep with the ARQ/ARQ genotype were chosen for this study. None of the selected animals bore additional polymorphisms in the fragment analyzed. The animals were free from the Brucella and Maedi-Visna viruses, were periodically vaccinated against enterotoxemia caused by *Clostridium perfringens* and were subjected to an antiparasitic treatment.

A total of 28 Rasa Aragonesa female sheep (infected and control group, aged 1–10 years) were included in this study (Table 1). In order to have specific comparison within groups, most of the scrapie infected animals were in the range of age of controls. For the gene transcription studies in different brain regions, 26 animals were used: 11 healthy control sheep, 10 sheep at the terminal stage of natural scrapie infection and 5 sheep with no clinical signs of infection (preclinical stage). Seventeen sheep were used for the protein immunodetection study: healthy control animals (*n* = 6), animals at the terminal stage of natural scrapie infection (*n* = 7) and a group of preclinical animals (*n* = 4). For the protein blotting assays, 12 animals were used divided into the three groups (healthy control, preclinical and clinical terminal stage animals), each group containing 4 animals. Table 1 shows the number and characteristics of the animals used in each study.

**Table 1:** Details of the analyzed animals used in immunohistochemistry (IHC), quantitative real-time PCR (qRT-PCR) or Western/dot blot

**Animals**	**Age (years)**	**Symptomatic status**	**IHC**	**qRT-PCR**	**Western/dot blot**
Ctrl1	6.5	Ctrl	✓	✓	-
Ctrl2	6	Ctrl	✓	✓	-
Ctrl3	9	Ctrl	✓	✓	✓
Ctrl4	10	Ctrl	✓	✓	✓
Ctrl5	3	Ctrl	✓	✓	✓
Ctrl6	5.5	Ctrl	✓	✓	✓
Ctrl7	4.5	Ctrl	-	✓	-
Ctrl8	4.5	Ctrl	-	✓	-
Ctrl9	4.5	Ctrl	-	✓	-
Ctrl10	1.5	Ctrl	-	✓	-
Ctrl11	2.5	Ctrl	-	✓	-
SC1	3.5	PC	✓	✓	✓
SC2	1.5	PC	✓	✓	✓
SC3	4.5	PC	✓	✓	✓
SC4	1	PC	✓	✓	✓
SC5	1.5	PC	-	✓	-
SC6	5.5	C	✓	-	-
SC7	6.5	C	✓	-	-
SC8	5	C	✓	✓	-
SC9	2.5	C	✓	✓	✓
SC10	4	C	✓	✓	✓
SC11	5.6	C	✓	✓	✓
SC12	7	C	✓	✓	✓
SC13	3.5	C	-	✓	-
SC14	4	C	-	✓	-
SC15	4.5	C	-	✓	-
SC16	4	C	-	✓	-
SC17	2	C	-	✓	-

Clinical status of each animal is described: control (Ctrl), preclinical (PC) or clinical (C).

The diagnosis of scrapie for clinical sheep was performed by describing the clinical signs associated with the disease [28]. The diagnosis of scrapie for clinical and preclinical animals was made by third eyelid [28] and rectal mucosa biopsies [29] and was confirmed in the medulla oblongata from the brain stem using a rapid test (Bio-Rad TeSeE) and immunohistochemistry to detect PrP^Sc^ using the 6H4 monoclonal antibody [30].

The control animals were matched for age, breed, sex and genotype and were selected from flocks belonging to scrapie-free regions. The third eyelid biopsy was carried out in all of the animals that were included in the study. The absence of PrP^Sc^ was also confirmed in the medulla oblongata, cerebellum, mesencephalon, thalamus, hypothalamus and frontal cortex by immunohistochemical methods (with the L42 antibody, described in the immunohistochemistry section) and by Western blotting (Prionics®-Check WESTERN).

### Necropsy and tissue collection

Necropsy was performed immediately after the animals were sacrificed by an intravenous injection of sodium pentobarbital and exsanguination. The postmortem examination of the animals did not reveal additional pathological findings. The tissue samples were rapidly preserved and processed according to the established guidelines regarding safety. The brain was sagittally divided into two halves, one of which was snap-frozen in liquid nitrogen prior to long-term storage at -80°C until RNA and protein extraction; the other half was fixed in 10% neutral-buffered formalin for 10 days, post-fixed and paraffin-embedded according to standard procedures for histopathological and immunohistochemical analysis. Later, the brains were dissected to isolate the most relevant neuropathological tissues, including the cervical spinal cord (cSc), medulla oblongata (Mobl), cerebellar cortex (Cc), thalamus (T) and hypothalamus (Ht). When the thalamus and hypothalamus are evaluated together, they are referred to as the diencephalon (Dien).

### Total RNA isolation and cDNA synthesis

Total RNA was isolated from Tissuemizer-disrupted cSc, Mobl, Cc and Dien using the RNeasy Mini Kit (Qiagen, Crawley, UK) according to the manufacturer’s instructions. To avoid genomic DNA contamination, the tissue samples were treated for 25 min at 37°C with two units of TURBO DNase (Ambion, Austin, TX, USA). The quality of the total RNA was assessed based on the presence of distinct intact 28S and 18S ribosomal RNA bands in an electrophoresis gel. The RNA concentration was determined by OD (260/280) using a Nanodrop spectrophotometer (UV spectrophotometer Q3000, Quawell Technology, Inc., USA). The complementary DNA (cDNA) was synthesized from 1 μg of RNA using random hexamers with the Superscript First Standard Synthesis System for RT-PCR (Invitrogen). To confirm the elimination of the genomic DNA, reverse transcription reactions were performed with and without the enzyme.

### Quantitative real-time PCR

Quantitative real-time PCR was performed to determine the expression of 6 genes (i.e., *CAPN6*, *COL1A2*, *COL3A1*, *GALA1, MTNR1B* and *MT2A*) in the four regions of study. The PCR primer sequences used for the quantification of the gene transcription have been previously described [20]. To improve the normalization accuracy, the geometric mean of three housekeeping genes was used to calculate a normalization factor (NF), which was used to normalize the expression level for each gene for each sample [31]. The NF was calculated from *GAPDH*, *G6PDH* and *RPL32* expression data. These are the three most stable reference housekeeping genes in the sheep medulla oblongata and have been used as internal references in previous expression studies of scrapie infection [32]. The primers and PCR conditions for the amplification of these housekeeping genes have been described previously [32,33].

The qRT-PCR method was performed using SYBR® Green (PE Applied Biosystems) assays. The PCR amplification was performed in an ABI-Prism Fast 7500 Sequence Detection System (PE Applied Biosystems). All qRT-PCR reactions were run in triplicate with 10-20 ng of cDNA as the template and a 300-nM final primer concentration in a total reaction volume of 10 μL. The universal conditions were used with an initial 10 min activation and denaturation step at 95°C, followed by 40 cycles of 15 s at 95°C and 30 s at 60°C. A dissociation curve protocol was used after each qRT-PCR reaction to identify the presence of nonspecific PCR bands or high levels of primer dimers. The baseline and threshold for the Ct calculations were set automatically with the ABI-Prism 7500 software, version 2.0.1, and the levels of gene transcription were determined using the comparative Ct method. A Student’s *t* test was used to determine if the differences observed between the groups were statistically significant (*P < 0.05 and **P < 0.01).

### Histopathology

After tissue fixation, the brain slices (4 mm) were immersed in 98% formic acid for 1 h to reduce prion infectivity. The tissues were then embedded in paraffin wax. The sections (4 μm) were stained with hematoxylin and eosin (HE) to evaluate the morphological changes. Additional sections were mounted on glass slides treated with triethoxysilyl propylamine for subsequent immunohistochemical procedures.

The brain structures analyzed in this study included the thalamus (T), hypothalamus (Ht), medulla oblongata (Mob), cervical spinal cord (cSc) and cerebellar cortex (Cc).

### Immunohistochemistry

PrP^Sc^ immunohistochemistry (IHC) in the five selected regions was performed as previously described [34]. Briefly, pre-treatment included immersion in formic acid for 15 min, treatment with proteinase K (4 μg/mL; Roche, Switzerland) for 15 min at 37°C and hydrated autoclaving. Monoclonal antibody L42 (1:500 dilution for 30 min; R-Biopharm, Germany) served as the primary antibody, EnVision™ (DAKO, Denmark) was utilized for visualization and 3,3’-diaminobenzidine (DAB) was used as the chromogen. In each case, pre-treatment before the application of these reagents involved heat-induced epitope retrieval with citrate buffer (pH 6.0).

Astrocytosis was evaluated based on glial fibrillary acidic protein (GFAP) immunostaining, as previously described [11,35]. Briefly, after heat-induced epitope retrieval pretreatment with citrate buffer (pH 6.0), the sections were incubated for 1 h at RT with rabbit polyclonal anti-GFAP antibody (1:400 dilution; DAKO). In routine immunoreactions, the omission of the primary antibodies in the control and scrapie slides served as negative controls.

Proteins encoded by the three upregulated genes (*CAPN6*, *GALA1* and *MT2A*) and the three downregulated genes (*COL1A2*, *COL3A1* and *MTNR1B*) were studied. To analyze the expression and distribution of these proteins, formalin-fixed, paraffin-embedded CNS tissue sections from the preclinical, clinical and control animals were studied by immunohistochemistry and were processed in a different manner for the different markers. The specificity for the ovine protein of interest was initially checked on the target ovine tissues with the chosen, commercially available antibodies. The optimal technical specifications for each antibody in ovine tissue are summarized in Table 2. The visualization system used was a 30 min incubation with a polymer linked to peroxidase and either anti mouse or anti rabbit immunoglobulin (EnVision, DAKO) followed by immersion in a 3,3’-diaminobenzidine solution with 0.05% hydrogen peroxide. Omission of the primary antibody was used as a control for nonspecific staining. The positive control tissues used for each antibody are specified in Table 2.

**Table 2:** Specifications for IHC in formalin-fixed and paraffin-embedded sheep brain tissue and Western/dot blot antibody dilution

			**IHC**				**Western/dot blot**
**Gene symbol**	**Description**	**Type/commercial reference**	**Dilution**	**epitope retrieval**	**Visualization system**	**Positive control**	**Dilution**
*COL1A2*	collagen 1 alpha 2	Mouse monoclonal against Collagen I Abcam AB6308	1:100	HIER Citrate buffer pH 6 10’ Pressure cooker Minimum 30’ cooling	DAKO EnVision anti mouse	Spleen (moderate perivascular and capsule staining, trabeculae unstained).	1:1500
*COL3A1*	collagen 3 alpha 1	Rabbit polyclonal against Collagen III Abcam AB7778	1:100		DAKO EnVision anti rabbit	Spleen (moderate perivascular staining and mild staining of trabeculae).	1: 5000
*GALA1*	galanin 1	Rabbit polyclonal ENZO Life sciences BML-GA1161-0100	1:400			Brain tissue (stains diffusely the neuropile of cerebellar molecular layer)	1:1000
*MTNR1B*	melatonin receptor 1b	Rabbit polyclonal Sigma SAB29002 12	1:100			Retina (stains the plexiform layers).	1:1500
*CAPN6*	calpain 6	Rabbit polyclonal Abcam AB76974	1:50	HIER Citrate buffer pH 6 20’ water bath at 95ºC Minimum 30’ cooling	DAKO EnVision anti rabbit	Placenta (stains placental epithelium).	1:1000
*MT2A*	methallothionein 2A	Mouse monoclonal against MT1+2 Dakocytomation M00639	1:200		DAKO EnVision anti mouse	Brain tissue (stains stellate shaped glial cells).	1:1500

The immunostained sections were examined with a NIKON Eclipse 90i optical microscope. The immunolabeling patterns on ovine brain tissue were described, and a semi-quantitative analysis of the immunolabeling intensity was performed. Immunolabeling scores, ranging from 0 (absence of immunolabeling) to 1 (mild), 2 (moderate), 3 (intense) and 4 (maximum intensity), were assigned to each brain region (i.e., cSc, Mobl, Cc, T and Ht). Each area was investigated as a global region for scoring. For the statistical analysis, the Mann Whitney test was applied (**P < 0.05 with a 95% confidence interval and *P < 0.1 with 90% confidence interval).

### Protein extraction and Western/dot blot analysis

In the four analyzed regions, at least 0.5 g of the brain frozen sections were homogenized in 5 mL of Prionics® Check Western homogenization buffer (Prionics AG, Zurich, Switzerland) and centrifuged at 10 000 × *g* for 10 min at 4°C. Supernatants containing total protein extracts were recovered and protein concentrations were measured by BCA (bicinchoninic acid) protein assay (Sigma-Aldrich, St. Louis, MO, USA). For Western-blot, after denaturation at 95 °C for 5 min, protein extracts (100 μg of total protein) were subjected to SDS/PAGE (12% and 8% polyacrylamide) at 150 V for 45 min and transferred to PVDF membranes (Bio-Rad, Hercules, CA, USA) at 200 V for 1 h using a Mini-PROTEAN 3 system (Bio-Rad, Hercules, CA, USA). For the dot blot technique, 30 μg of total protein were deposited in duplicate over the PVDF membranes.

The PVDF membranes were treated with blocking solution (TBS buffer, 0.5% Tween 20 and 5% non-fat milk) for 1 h at RT (Room Temperature), and then incubated for 1 h with the appropriate primary antibody diluted in blocking buffer. The primary antibodies used for immunoblotting assays were the same used in IHC staining experiments (Table 2). Next, the membranes were incubated for 1 h with HRP-conjugated secondary antibody diluted 1:3500 in blocking buffer (goat anti-mouse IgG-HRP for anti-COL1A2 and anti-MT2A or goat anti-rabbit IgG-HRP for anti-CAPN6, anti-COL3A1, anti-GALA1 and anti-MTNR1B; Santa Cruz Biotechnology). Three washes 10 min each with TBS-0.5% Tween 20 were performed between incubation periods. Western/dot blots were developed using an ECL + reagent kit (Amersham-GE Healthcare) and visualized with a Bio-Rad VersaDoc imaging system. For the purpose of these studies, blots were imaged following exposure for 10 min (enhanced images) to ensure that samples containing comparatively low protein concentrations were detectable.

Western blot results were used to determine the specificity of the used antibodies. The quantification analysis was performed based on the dot blot results using the ImageJ 1.4.3.67 image-analysis software package (Psion Image, NIH) following a simple method of analysis, performed by integrating the grey levels of pixels (volume) surrounded by a circular selection. This method is described on the ImageJ website [36]. Density of immunoreactive dots was normalized for Ponceau Red and is reported as arbitrary units (a.u.). Data are expressed as means ± standard error. A Student’s *t* test was used to determine if the differences observed between the groups were statistically significant (*P < 0.05 and **P < 0.01).

## Results

### Scrapie-associated neuropathology

**Figure 1:**
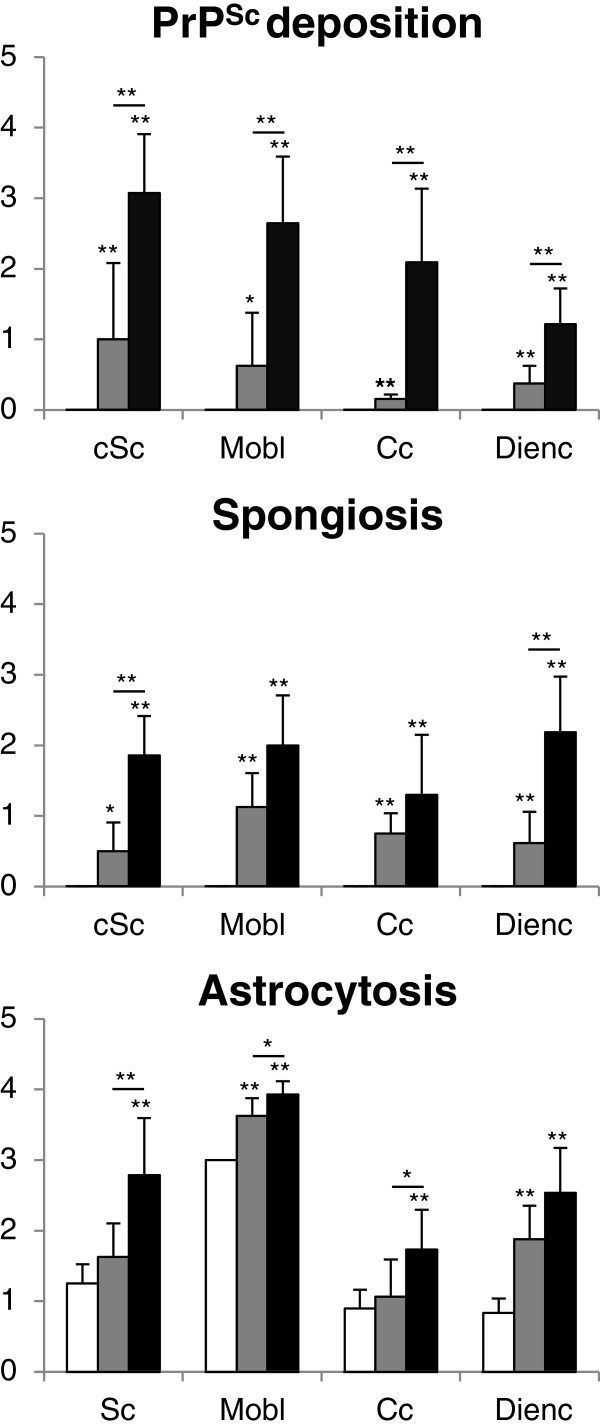
**Semi quantitative assessment values of PrP**^**Sc **^**deposition, spongiform degeneration and glial fibrillary acidic protein expression as a marker for astrocytes in the spinal cord, medulla oblongata, cerebellum and diencephalon of control (white bars)**, **preclinical (grey bars) and clinical (black bars) scrapie sheep evaluated (from 0: negative, to 4: lesion/staining present at maximum intensity).** Significant differences were determined using the Mann Whitney test (**P < 0.05 and *P < 0.1).

Prionopathy associated spongiosis, PrP^Sc^ deposition and gliosis (assessed by GFAP immunoreactivity) were evaluated semiquantitatively in the four analyzed brain regions (cSc, Mobl, Cc and Dien) from 6 controls, 4 preclinical and 7 clinical scrapie-infected sheep (Figure 1). The distribution of these markers was consistent with most of the features previously described for classical scrapie [18]. Despite the high variability observed for all markers within the scrapie groups, in most cases, statistically significant differences were recorded between these animals and the control group (*P* < 0.05).

PrP^Sc^ deposition and spongiform degeneration were detected only in the infected animals, and the absence of PrP^Sc^ was characteristic of the control group. Regarding the clinical animals, intraneuronal and neuropil PrP^Sc^ immunolabeling was strong in cSc and Mobl, and moderate in Cc and Dien, which was in accordance with classical scrapie infected sheep. In preclinical animals, PrP^Sc^ deposits were moderate in Sc and Mobl and weak in Cc and Dien, showing a similar distribution to that of clinical sheep. In summary, the presence of PrP^Sc^ and spongiform degeneration was lower in preclinical than in clinical sheep. The evaluation of haematoxylin-eosin-stained sections revealed a significant and moderate to weak increase of spongiosis in the Mobl, Cc, cSc and Dien of the preclinical group (Figure 1). Spongiosis was also shown in the animals with a clinical state of scrapie, in which the lesion was significantly stronger in the Dien following by Mobl and cSc. The spongiosis scores were moderate in the Cc of clinical animals compared with controls.

A generalized increase of astroglial marker glial fibrillary acidic protein (GFAP) immunostaining was observed in the brains of the scrapie-infected sheep. Hyperplasia and hypertrophy of the stellate GFAP-positive cells, consistent with reactive astrocytosis, was observed in all analyzed brain regions. For the clinical group, the Dien and cSc sections evaluated show higher scores, followed by moderate scores in Mobl and Cc. Regarding the preclinical group, only Mobl and Dien show a moderate and significant increase of GFAP deposition (Figure 1).

### Gene transcription

**Figure 2:**
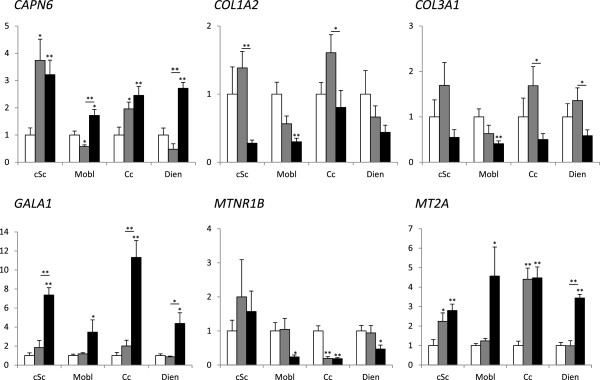
**Gene transcription profiles of six scrapie-related genes (i.e., *CAPN6*, *COL1A2*, *COL3A1*, *GALA1*, *MTNR1B *and *MT2A*) in four CNS regions in control (white bars), preclinical scrapie (grey bars) and clinical scrapie (black bars) sheep in the cervical spinal cord, medulla oblongata, cerebellar cortex and diencephalon.** Differences between groups were analyzed using a Student’s *t*-test *P < 0.05 and **P < 0.01).

The expression of the six genes was analyzed in four different regions of the central nervous system (i.e., the cSc, Mobl, Cc and Dien) in the clinical and preclinical scrapie-infected and control sheep. As shown in Figure 2, we confirmed the overexpression of *CAPN6*, *GALA1* and *MT2A* in the medulla oblongata in clinical stage scrapie-infected animals, as well as the downregulation of *COL1A2*, *COL3A1* and *MTNR1B.* In general, this profile was maintained in the other CNS regions of clinical sheep. Upregulation of *GALA1* was notable in the cervical spinal cord and the cerebellum (8- and 12-fold change [FC], respectively) and, to a lesser degree (4-FC), in the diencephalon. A significant increase of *MT2A* and *CAPN6* (2-4-FC) was observed in all the analyzed brain regions; and a 5-fold decrease of *MTNR1B* was observed in the cerebellum. Although a trend of *COL1A2* and *COL3A1* downregulation was observed in all brain regions, their expression levels were only significantly different from controls in the medulla oblongata.

In contrast, the preclinical animals did not present significant changes with respect to controls in most cases. However, the upregulation observed for *CAPN6* and *MT2A* in the clinical animals was also observed in the preclinical spinal cords and cerebellar cortex, and the downregulation of *MTNR1B* was also evidenced in the preclinical cerebellar cortex. In contrast, *CAPN6* in the medulla oblongata displayed opposite and significant (*p* < 0.05) gene transcription profiles compared to those observed in the clinical phases of the disease.

### Protein distribution in central nervous system

Regarding immunohistochemical markers, the first obstacle that needed to be overcome was the lack of commercially available ovine-specific antibodies against most of the selected genes and the lack of previously published information on the immunolabeling that should be expected for such markers in the adult ovine CNS. A number of combinations of epitope retrieval treatment and antibody dilution were tested to determine the optimal immunostaining procedures.

A detailed description of the immunostaining pattern for each antibody is provided because no previous data, to the authors’ knowledge, has been published on the immunolabeling of the proposed markers in the ovine CNS, except for MT1 + 2 [11].

#### Collagen 1 alpha 2 immunolabeling

In the ovine skin samples, the antibody against COL1A2 stained the subepithelial connective tissue and, in the spleen, a mild perivascular signaling as well as in the capsule was observed but the trabeculae were negative.

**Figure 3:**
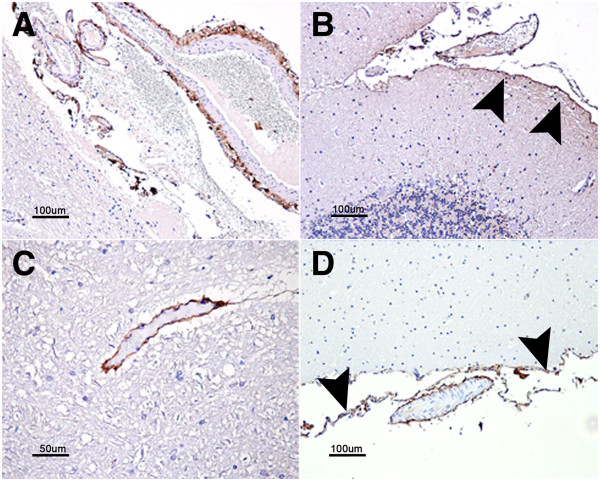
**Immunostaining patterns of COL1A2 (A and B) and COL3A1 (C and D).** COL1A2 perivascular staining in the meninges (**A**) as well as leptomeningeal cells (arrowheads in **B**). COL3A1 stained medium sized blood vessel walls within the nervous parenchima (**C**) as well as leptomeningeal cells (arrowheads in **D**) and meningeal blood vessels (**D**).

COL1A2 staining in the CNS was confined to leptomeningeal and perivascular cells but was very mild or absent in medium- or small-sized intraparenquimatous vessels (Figure 3A and B). No significant differences were observed when comparing the control and scrapie-infected animals.

#### Collagen 3 alpha 1 immunolabeling

In the ovine skin samples, COL3A1 antibody stained the subepithelial connective tissue and, in the spleen, a mild perivascular immunostaining as well as in the trabeculae was observed, but the capsule was negative.

In the CNS, perivascular labeling corresponding to the basement membrane of blood vessels was observed. The amount of stained intraparenquimatous blood vessels was slightly higher in the cervical spinal cord compared to other brain regions studied. Additionally, the leptomeninges were labeled with the COL3A1 antibody (Figure 3c and d). Again, no significant differences were observed between the control and scrapie-infected animals.

#### Galanin 1 immunolabeling

**Figure 4:**
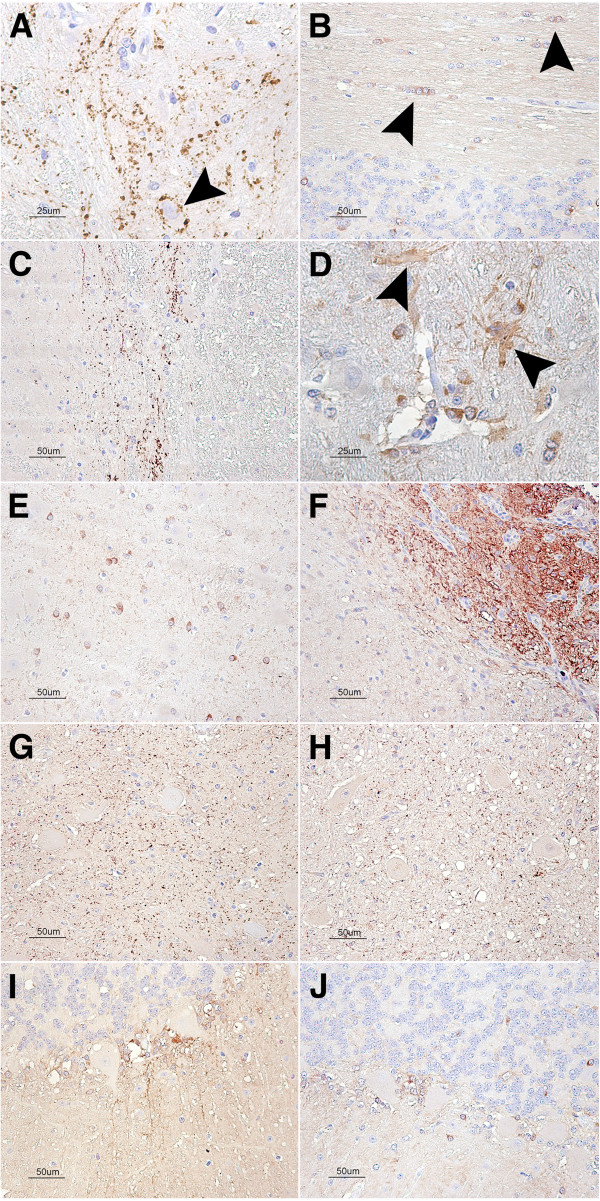
**GALA1 patterns. Galanin immunostaining patterns in different CNS regions of control animals (A to F).** (**A**) Synaptic like punctiform pattern in the medulla oblongata, note the perineuronal arrangement (arrowhead). (**B**) Cytoplasmic immunostaining of oligodendrocyte rows in the cerebellar white matter (arrowheads). (**C**) Synaptic pattern in the cervical spinal cord dorsal horn (*Substantia gelatinosa*). (**D**) Cytoplasmatic immunostaining of astrocytes in the mesencephalon. (**E**) Intracytoplasmic immunostaining in multiple cells in the Thalamus, either glial cells or small neurons. (**F**) The neurons and glial cells in the area postrema are strongly immunostained. (**G**) GALA1 immunolabeling in the medulla oblongata in the control animals (**H**) clinical scrapie-infected animals. (I) GALA1 immunolabeling in the cerebellar cortex in the control animals (**J**) clinical scrapie-infected animals. GALA1 immunolabeling was slightly more intense in the cerebellar cortex on the clinical scrapie-infected sheep compared to the control. The remaining regions show little variation.

A punctiform, synaptic-like immunostaining was observed in several regions, such as the nucleus of the solitary tract in the medulla oblongata, which occasionally displayed a perineuronal arrangement (Figure 4A), or in the *substantia gelatinosa* (Figure 4C) in the dorsal horn of the cervical spinal cord. In addition, multiple cells exhibited intracytoplasmic immunolabeling, particularly in the hypothalamus, but also in the cerebellar cortex, thalamus, mesencephalon and medulla oblongata (Figure 4E). The region postrema was strongly stained (Figure 4F). The immunostaining in the cerebral cortices was minimal. Some of the stained cells could clearly be identified morphologically as glial cells, either oligodendrocytes (Figure 4B) or astrocytes (Figure 4D), while the majority of the cells were identified as medium- to small-sized neurons. Occasionally, punctiform intracytoplasmic immunostaining was observed in larger neurons, such as the motor neurons of the cervical spinal cord’s ventral horn. In the cerebellum, these small, positively stained cells were located mainly in the Purkinje cell layer, around the piriform neurons, which occasionally had neurites decorated with punctiform immunolabeling (Figure 4I).

When comparing the uninfected animals with those infected with scrapie, minimal differences were observed. Increased immunolabeling was noticed in the scrapie-infected sheep, particularly in the medulla oblongata and in the cerebellar cortex, mainly due to an increase in the immunostaining of glial-shaped cells (Figure 4H and J). Semiquantitative evaluation of the signaling yielded statistically significant differences between the preclinical and clinical groups in the cerebellum.

#### Calpain 6 immunolabeling

**Figure 5:**
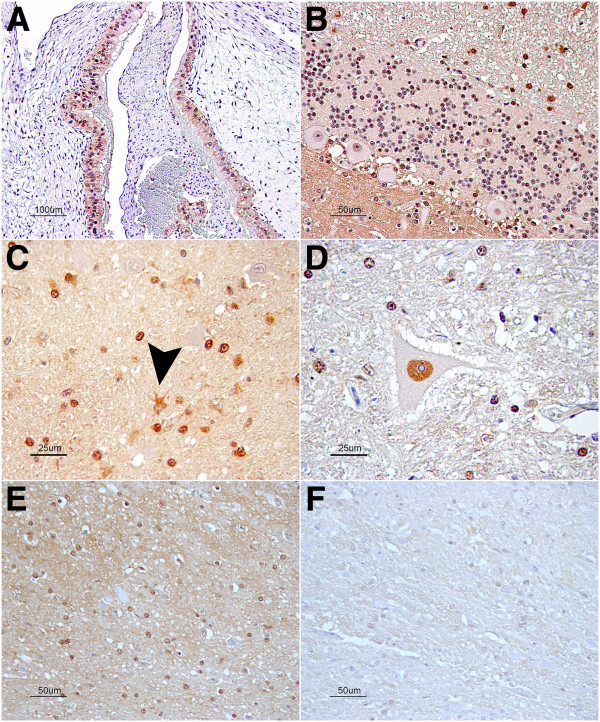
**CAPN6 immunostaining.** (**A**) Staining of the sheep placental epithelium as a positive control for the technique. (**B**) Cerebellar cortex: moderate, diffuse neuropil staining of the gray matter of the molecular layer; neuronal cytoplasm was unstained. (**C**) Thalamus: in the white matter, the glial cells were strongly immunolabeled. Mainly, it consisted of nuclear staining, but the cytoplasm was occasionally observed (arrowhead). (**D**) Ventral horn of cervical spinal cord: Intranuclear staining was also evident in motor neurons. (**E**) Dorsal horn of a scrapie clinical case in which staining was stronger than in the (**F**) same region of a control sheep.

Sheep placental tissue was successfully stained (Figure 5A) and included in the experiment as a positive control because *CAPN6* expression has been described in the placental epithelium in other species [37]. CAPN6 immunostaining of the sheep brain parenchyma consisted of a moderate diffuse neuropil staining that was more intense in some of the brain regions studied, such as the molecular layer of the cerebellum (Figure 5B), the *substantia gelatinosa* of the dorsal horn of the cervical spinal cord and the medulla oblongata. The neuronal cytoplasm was generally unstained, but the nuclei were positive (excluding the nucleolus) (Figure 5D). In addition, strong glial cell staining was observed in the white matter, primarily in the nucleus but also in the cytoplasm (Figure 5C).

**Figure 6:**
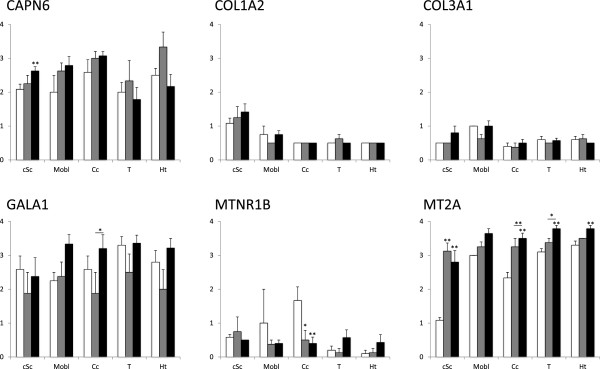
**Semiquantitative assessment values of CAPN6, COL1A2, COL3A2, GALA1, MTNR1B and MT1+2 IHC evaluated (from 0: negative, to 4: staining present at its maximum intensity) in the spinal cord, medulla oblongata, cerebellum, thalamus and hypothalamus.** White bars: control sheep, grey bars: preclinical scrapie-infected and black bars: clinical scrapie-infected sheep. Significant differences between groups were determined using the Mann Whitney test (**P < 0.05 and *P < 0.1).

The variability observed between different individuals upon quantification was considerable. However, both scrapie-infected groups tended to show higher scores in the cerebellum, medulla oblongata and cervical spinal cord. This increase was statistically significant when the cervical spinal cord score was compared between the control and clinical scrapie groups (Figure 6).

#### Melatonin receptor 1B immunolabeling

**Figure 7:**
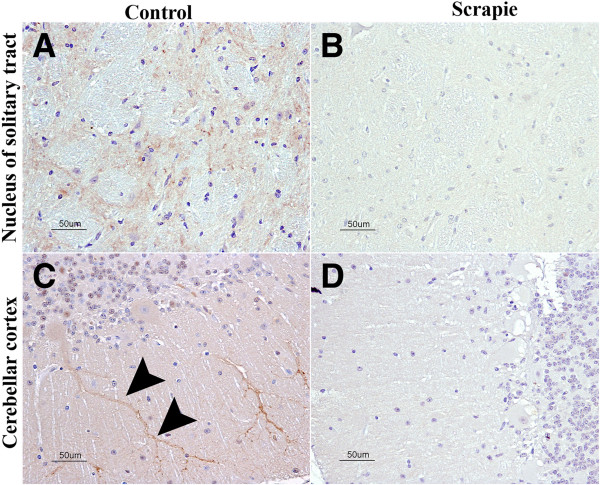
**MTNR1B immunostaining.** (**A**) In the nucleus of the solitary tract of the medulla oblongata, mild, diffuse neuropil staining was observed and was stronger in the control animals than in the (**B**) scrapie-infected animals. (**C**) In the molecular layer of the cerebellar cortex, a strong signal was observed in the Purkinje cells and their dendrites (arrowheads). (**D**) This signal was reduced or absent in the scrapie-infected animals.

The ovine retina was used as a positive immunohistochemical control. In this tissue, both the external and internal plexiform layers were stained positively by the antibody against MTNR1B. In the CNS, a mild, diffuse neuropil staining was observed in the gray matter, but the white matter was negative. This staining was particularly intense in the dorsal horn of the cervical spinal cord. In the cerebellar cortex, the Purkinje cell dendrites were occasionally observed with intense cytoplasmic immunolabeling (Figure 7C).

When the control animals were compared to the scrapie-infected animals, no significant differences were observed regarding MTNR1B immunostaining in any of the CNS regions studied, with the exception of the cerebellum (Figure 7C and D). A significant reduction of the Purkinje cell dendrite staining was noticed between the control and preclinical sheep (*P* < 0.1) and between the control and clinical sheep (*P* < 0.05) (Figure 6). The medulla oblongata also showed a stronger staining intensity in the controls compared to the scrapie-infected animals; however, the limited availability of this region for immunohistochemical studies precluded statistical significance (Figure 7A and B).

#### Metallothionein 1 + 2 immunolabeling

The specific immunolabeling patterns of MT1 + 2 antibodies in the sheep brain have been published [11]. Briefly, strong intracytoplasmic glial staining was observed in all studied brain regions.

A semiquantitative analysis shows statistically significant higher scores in scrapie-infected animals in almost all the regions evaluated when compared to the control group. Differences were also observed between the clinical and preclinical groups, particularly in the thalamus and cerebellum (Figure 6).

### Protein quantification by blotting assay

**Figure 8:**
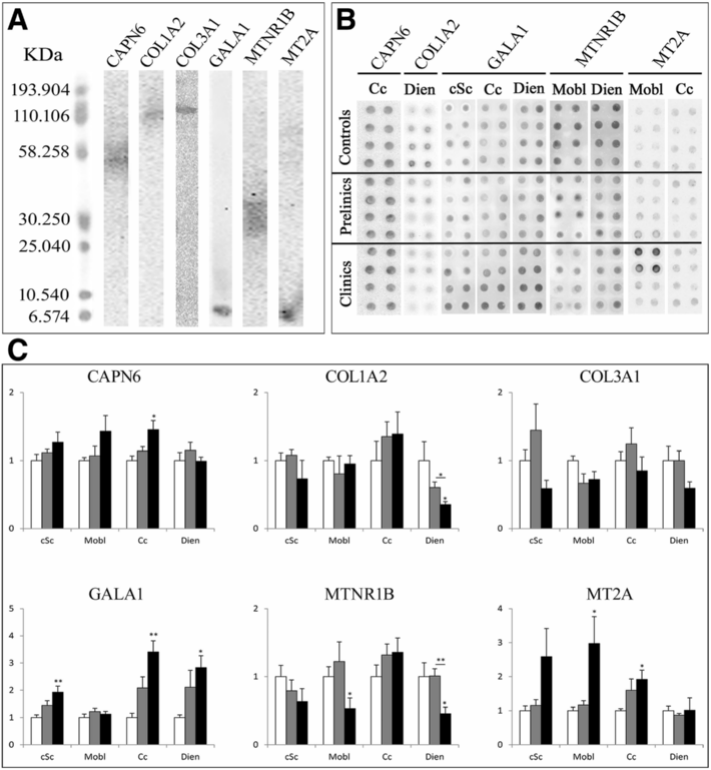
**Western-Blot and dot blot Quantification of protein expression results in scrapie CNS tissues.** Specificity of anti-CAPN6, anti-COL1A2, anti-COL3A1, anti-GALA1, anti-MTNR1B and anti-MT2A antibodies in the ovine medulla oblongata, as detected by Western blotting (**A**). Images of significant results of dot blot quantification (**B**). Protein expression profiles of the six scrapie-related proteins (i.e., CAPN6, COL1A2, COL3A1, GALA1, MTNR1B and MT2A) in four CNS regions in control (white bars), preclinical scrapie (grey bars) and clinical scrapie (black bars) sheep in the cervical spinal cord, medulla oblongata, cerebellar cortex and diencephalon using the dot blot assay (**C**). Density of immunoreactive dots was normalized for Ponceau Red and reported as arbitrary units (a.u.). Data are expressed as means ± standard error. Differences between groups were analyzed using a Student’s *t*-test (*P < 0.05 and **P < 0.01).

For the immunoblotting assays and in order to determine the optimal procedures, many dilution combinations of the primary and secondary antibodies were tested. Western blot results displayed low band intensities, meaning low expression of those proteins in CNS. For better immunoblotting quantification we performed dot blot assays. In contrast, Western blot results show unique and specific bands for all the analyzed proteins (~70 kDa, ~130 kDa, ~140 kDa, ~8 kDa, ~40 kDa and ~7 kDa for anti-CAPN6, anti-COL1A2, anti-COL3A1, anti-GALA1, anti-MTNR1B and anti-MT2A, respectively) (Figure 8A). Figure 8 presents the dot images for the significant results (Figure 8B) and the protein quantification results of the six chosen proteins in cSc, Mobl, Cc and Dien (Figure 8C). The significant changes of protein expression profiles were in agreement with the variations observed at the transcript level. These significant differences were only observed in the clinical scrapie group.

Proteins that presented significant changes in their expression data in clinical sheep were CAPN6 in the Cc region, GALA1 in cSc, Cc and Dien, MTNR1B in Mobl and Dien regions and MT 1 + 2 in Mobl and Cc regions.

## Discussion

The molecular mechanisms of prion disease pathology are still poorly understood. Our group developed a genomic study assessing the medulla oblongata of sheep naturally infected with scrapie in the clinical stage [20], and we studied a set of late phase, scrapie-related genes. Only one of these genes, *MT2A*, had previously been reported to be associated with prion diseases. The other markers (i.e., *CAPN6*, *COL1A2*, *COL3A1*, *GALA1* and *MTNR1B*) belong to gene families with a known role in other neurodegenerative diseases, such as Alzheimer’s or Parkinson’s disease in humans [21-26,38,39].

Genomic analyses can identify prion-related biomarkers or candidate targets for new therapies. However, the results obtained from these studies must be validated in different animals, brain regions and clinical phases of the disease. Three methodologies have been combined in the present work, qRT-PCR, immunohistochemistry and Western/dot blot, to study in depth the possible role of these markers in prion-induced neuropathology. We analyzed the gene transcription profiles for the 6 genes in four CNS regions for a large set of scrapie-infected sheep (*n* = 15) in the early and terminal stages of natural scrapie. We investigated the resulting protein level changes and the distribution of those proteins in five brain regions: the cervical spinal cord, medulla oblongata, cerebellar cortex, thalamus and hypothalamus.

In order to discard other factors with possible interference with other diseases, we selected animals without any apparent pathology other than scrapie. In addition, the study design contrasted the amount of infected animals versus controls, in order to minimize other factors that could also influence the results.

We present here the first published description of the CAPN6, COL1A2, COL3A1, GALA1 and MTNR1B immunostaining profiles in the adult sheep brain, as well as their modification upon scrapie infection.

The extracellular matrix (ECM) in the central nervous system is enriched in proteoglycans (mainly lecticans), glycoproteins (e.g., tenascin) and hyaluronic acid [40,41]. Collagen I and III molecules, which are abundant in the ECM of other tissues, are mainly restricted to the vascular and meningeal compartments of the central nervous system. In agreement with our earlier work [20], a significant decrease in the expression levels of *COL1A2* and *COL3A1* was observed in the medulla oblongata in sheep at the clinical stage of scrapie infection.

It has been suggested that changes in cerebrovascular organs or in the brain blood barrier in response to prion insult might be occurring in scrapie-infected brains. A decrease in collagen IV is observed in Multiple System Atrophy [42], and the reduction of COL6A1 has been reported in AD [43]. In contrast, the overexpression of the collagenous type II transmembrane protein (COL25A1) leads to AD-like pathology and different collagens, including COL1A1 and COL3A1, are induced in multiple sclerosis lesions [44]. The expression of COL1A2 transcripts was strongly downregulated in the medulla oblonga of scrapie animals both here and in our previous study, suggesting a role in prion neuropathology since this region includes a circumventricular organ, the area postrema, which is enriched in different collagen types and one of the potential entrances of the prions into the brain [45]. However, neither the immunohistochemical nor the dot blot analysis confirmed this variation at the protein level. Expression of the collagen genes (COL1A1, COL1A2, and COL3A1) is regulated at the transcriptional [46] and post-transcriptional [47] levels and several RNA-binding proteins may act as stabilizers of the collagen mRNA [48]. A post-transcriptional regulation could be acting in scrapie avoiding the loss of collagen proteins in the damaged brain. Then, our results warn about using these molecules as potential biomarkers for therapies or preclinical diagnosis.

Galanin is an inhibitory neuropeptide to which a neuroprotective role has been attributed [49,50]. However, increased galanin-1 levels have also been suggested to impair cognitive function [25], and *GALA1* has been found to be overexpressed in Alzheimer’s disease [21,22]. *GALA1* is overexpressed in the medulla oblongata of clinical scrapie-infected sheep [20]. In the present study, *GALA1* gene transcription was significantly increased in all four analyzed brain regions of the clinical scrapie-infected sheep. Immunoblotting protein quantification revealed nearly the same changes as those observed in gene transcription. Previous studies have reported neuronal immunostaining of galanin in the sheep hypothalamus [51-53], but little to no information was found regarding glial labeling or other CNS regions. In other species, such as rats and humans, galanin immunolabeling has been reported not only in neurons but also in astrocytes and oligodendrocyte precursors [54]. Galanin is enriched in the hypothalamus, *locus ceruleus*, amygdala, bed nucleus of the *stria terminalis*, dorsal raphe nucleus, dorsal root ganglia and cervical spinal cord [49]. The distribution of galanin immunolabeling observed in sheep was similar to the descriptions for other species but, in addition to the known brain regions, we provide the first description of its presence in the cerebellar cortex. The role of galanin in neurodegenerative conditions is still unclear and deserves further attention. Our results show increased galanin expression in scrapie-infected animals, particularly in the cerebellar cortex of end-stage animals. It could be hypothesized that a relationship may exist between these changes in galanin expression and the clinical signs of scrapie, such as ataxia. In accordance to this, the clinical animals showing the highest GALA1 protein expression also displayed the highest scores for PrP^Sc^ deposition and spongiosis (data not shown).

The expression of this protein was not significantly modified in preclinical sheep avoiding its use as a potential biomarker for preclinical animals. Nevertheless, once more, the relevance of this marker in prion pathogenesis has been confirmed, and GALA1 could be considered a potential therapeutic target for the treatment of TSE.

Calpains are intracellular, non-lysosomal, calcium-dependent, cysteine proteases that are involved in proteolysis, apoptotic cell death, necrosis and other physiological events. Calpain 6 is known to bind and stabilize microtubules in the regulation of microtubule and cytoskeletal organization during embryonic development [55,56]. In consequence, CAPN6 expression should be downregulated after birth, except for placenta and for uterine cell tumors [57]. Since Calpain 6 colocalizes with microtubules, an immunolabeling restricted to the cytoplasm was expected in the brain. The described intraglial and neuropile staining fit well, but the intranuclear staining observed would be difficult to explain, although previous publications also report nuclear staining, albeit in other tissues [57]. The different forms of calpains localize in a different manner, whereas μ-calpain is present mainly in the somatodendritic compartment of neurons, with lower levels in the myelinated axons; m-calpain is located mainly in axons and also in myelin [58]. The immunolabeling pattern described in sheep brain in this report was in accordance with those described for μ-calpain and m-calpain.

In addition to our previous study showing the overexpression of the Calpain 6 gene in the medulla oblongata of terminal scrapie [20], other members of the calpain family have been associated with prion diseases [59,60] and other neuropathological events, contributing to Alzheimer’s [61-63] and Parkinson’s [64]. The present study shows a statistically significant increase in gene transcription of *CAPN6* in all the analyzed brain regions (i.e., cSc, Mobl, Cc and Dien) of the clinical animals and in the cSc and Cc of the preclinical animals, which was in accordance with the increase in calpain expression throughout the course of the disease in mice [60].

In scrapie affected sheep, astrocytosis is one of the most common described histopathological events related with the disease [65]. Thus, glial activation, which is particularly strong in the Mobl and cSc, might partially explain the CAPN6 expression changes.

In agreement with the gene transcription results, a tendency for increased calpain 6 protein immunolabeling and immunoblotting was observed in the majority of the regions analyzed in scrapie-infected animals, being particularly significant in the cSc and Cc of clinical cases. Further attention should be given to the involvement of calpain 6 in the pathogenesis of scrapie because the neuropil immunostaining was increased in clinical scrapie animals, particularly in the dorsal horn of the spinal cord. This region includes sensory pathways (Substantia gelatinosa and proprius nuclei are craneally connected with the spinal tract of the trigeminal nerve) and is strongly involved in nociception (tactile information), thus changes at this level could be related to some of the scrapie clinical signs, such as hyperesthesia, pruritus and scratch response. Our results suggest that Calpain 6 may be a potential therapeutic target in prion diseases as they are in Parkinson’s disease [66]. This suggestion is supported by other studies describing that calpain-mediated endoproteolytic cleavage of PrP^Sc^ may be an important event in prion propagation [67]. However, the possible involvement of calpain-like activity in the normal processing of PrP^C^[68] has to be considered in any further therapeutic study focused on a possible regulation of this target. In addition, since this protein is overexpressed in the Sc and Cc of preclinical individuals, we could propose it as a good candidate diagnostic biomarker for scrapie.

The indoleamine melatonin (*N*-acetyl-5-methoxytryptamine) is synthesized in the pinealocytes, located in the pineal gland (*Epiphysis cerebri*) of the mammalian brain. In addition to its role as a chronobiotic in mammals, melatonin is an antioxidant and an effective protector of mitochondrial bioenergetics [69]. Melatonin protects from Aβ toxicity, especially at the mitochondrial level [70,71], and is used as an antioxidant in AD patients [72]. All known actions and effects of melatonin are mediated by membrane receptors on the cell surface (i.e., MTNR1A and MTNR1B) [73,74]. Expression of the human MTNR1B (also called MT2 receptor) protein was demonstrated in the hippocampus and cortex by immunohistochemical studies, reporting decreased intensity of the MTNR1B staining in AD cases [75,76]. In our study, the expression of the *MTNR1B* gene was significantly downregulated in the Mobl, Cc and Dien regions of clinical sheep and in the Cc region of the preclinical scrapie-infected animals. This finding was consistent with the significantly lower MTNR1B protein expression in Mobl and Dien in clinical animals, detected by dot blot quantification, and the significantly lower score of MTNR1B immunostaining in the cerebellum of clinical and preclinical animals. Our results strongly suggest the contribution of the melatonin receptor in ovine classical scrapie pathology, even in the early phase of the disease and in different brain regions; therefore, melatonin may be a good candidate target for future prion disease therapies. In accordance with our suggestion, a recent report has shown that melatonin-induced autophagy protects against prion protein-mediated neurotoxicity [77].

MT 1 and 2 are cell stress-related genes which are coordinately regulated by metals, glucocorticoids and inflammatory stress signals [78]. In addition to the overexpression previously reported for *MT2A* in the medulla oblongata during clinical scrapie [20], a significant over expression of *MT2A* was observed in the four CNS regions analyzed in clinical scrapie and in the cSc and Cc of the preclinical scrapie-infected animals. The up-regulation of MT2 was confirmed by immunohistochemistry and immunoblotting assay. These results were in agreement with the previous descriptions of metallothioneins in experimental and natural scrapie and other TSE [38,39,79-81]. Those reports suggest that oxidative stress, most likely a consequence of the glial activation elicited by the PrP^Sc^ deposition, is a pivotal mechanism in prion neurodegeneration pathogenesis. In addition, the confirmed upregulation of metallothioneins during preclinical natural scrapie suggests that these proteins are possible good target candidates for prion disease biomarkers.

In summary, we present here the gene transcription data and immunohistochemical protein distributions for CAPN6, COL1A2, COL3A1, GALA1, MTNR1B and MT2A in various regions of the CNS based on a large set of sheep with naturally occurring classical scrapie, including both the clinical and preclinical stages of the disease. The CNS distribution of five of these proteins, as well as their correlation with the gene transcription, is reported here for the first time. The increased expression of *CAPN6*, *GALA1* and *MT2A* in most of the brain regions analyzed suggests a potential role of these genes in early and terminal scrapie pathogenesis, which is further supported by immunohistochemical and immunoblotting evidence. These proteins would be potential biomarkers for preclinical diagnosis of scrapie because they have been detected in young animals, however, further analysis studying the expression of these markers in peripheral tissues is necessary. Also these markers could be considered potential therapeutic targets for prion diseases, since they are used in other neurodegenerative diseases. The down-regulation of *MTNR1B* argues in favor of a loss of the neuroprotection produced by melatonin, particularly in the cerebellar cortex region. These genes can be considered good candidate targets for the development of future prion therapies.

## Competing interests

The authors declare that they have no competing interests.

## Authors’ contributions

HF and EV performed the experiments and drafted the manuscript. RB conceived of the study, and participated in its design and coordination and drafted the manuscript. MM participated in the IHC study. PM participated in the discussion of IHC results. AV participated in the discussion of IHC results and helped to draft the manuscript. MP participated in the assessment of IHC results and protein distribution and helped to draft the manuscript. IMB participated in the design of the study, participated in the molecular genetics and protein expression studies and drafted the manuscript. JJB participated in its design and coordination and drafted the manuscript. All authors read and approved the final manuscript.
